# Supplementary material for: Drosophila melanogaster as a model for studies related to the toxicity of lavender, ginger and copaiba essential oils
Source: PLoS One. 2023 Sep 28;18(9):e0291242. doi: 10.1371/journal.pone.0291242 (PMC10538661; doi:10.1371/journal.pone.0291242)
Supplement: S1 Dataset — Chromatographic analysis of the copaiba essential oil utilized in this study, including the lot number, chromatographic chart, and a table of the constituents of the oil. (PDF) [file pone.0291242.s001.pdf]

Aromatic Plant Research Center

We provide uncompromising
quality control for your products.

Customer : doTERRA International
Lot Number : 202615
Date Filled : 09/17/2020

Column : ZB5 (60 m length × 0.25 mm inner diameter × 0.25 μm film thickness)
Instrument : Shimadzu GCMS-QP2010 Ultra
Carrier gas : Helium 80 psi
Temperature ramp : 2 degrees celsius per minute up to 260-degrees celsius
Split ratio : 30:1
Sample preparation : 5%w/v solution with Dichloromethane.

Comments:
The analysis of this Copaiba lot revealed no contaminants or adulteration.
The sample meets the expected chemical profile for authentic essential oils of Copaifera species.

Analysied by : Ambika Poudel
Reviewed by : Dr. Prabodh Satyal

Aromatic Plant Research Center
230 N 1200 E STE 100
Lehi, UT 84043
www.aromaticplant.org

Copaiba Essential Oil

Customer : doTERRA International

Lot Number : 202615

Date Filled : 09/17/2020

Chromatogram

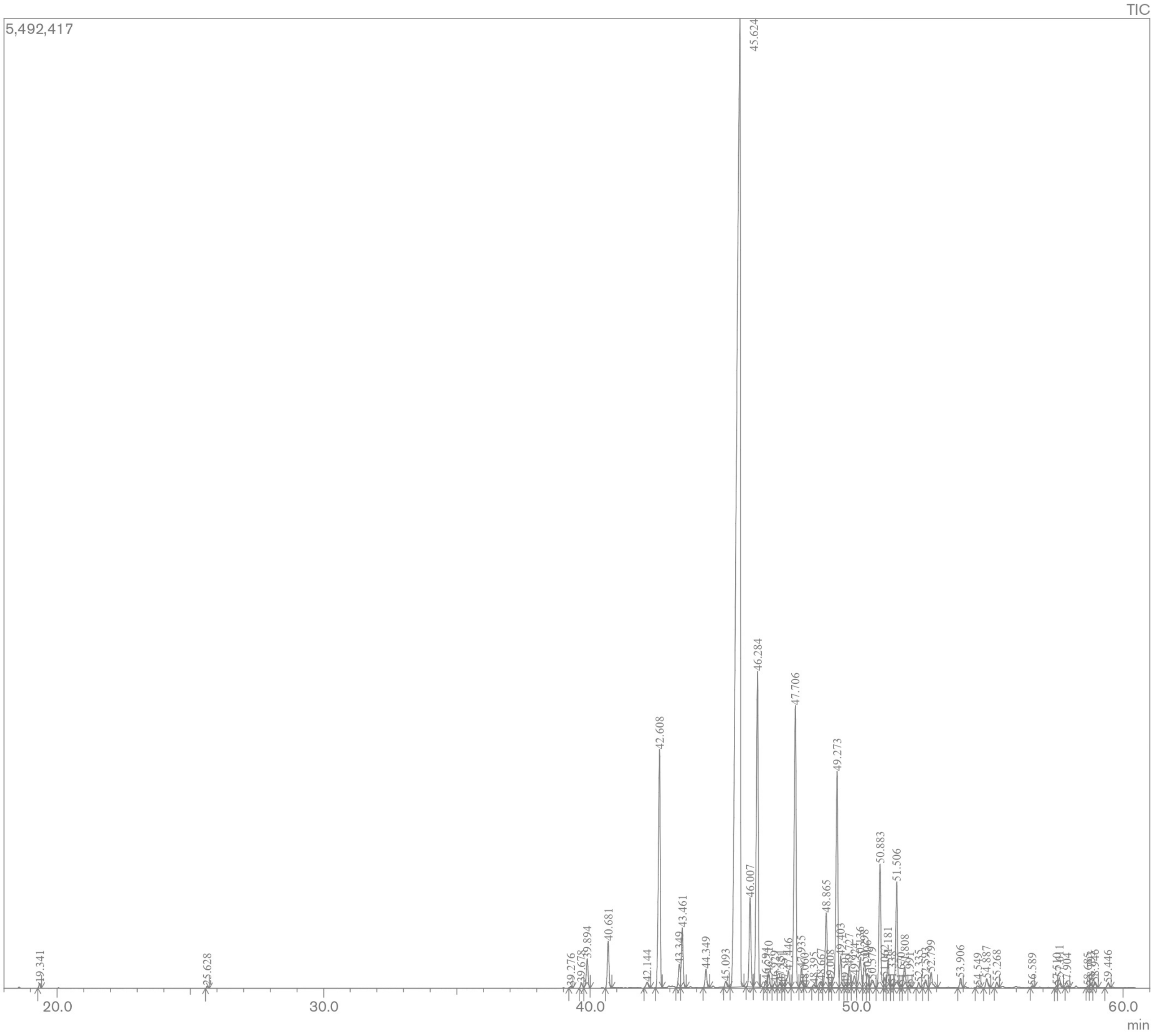

Aromatic Plant Research Center
230 N 1200 E STE 100
Lehi, UT 84043
www.aromaticplant.org

Peak Report

Peak#	R.Time	Name	Area%
1	19.341	cis-beta-Ocimene	0.09
2	25.628	allo-Ocimene	0.04
3	39.276	Bicyclo nonane	0.03
4	39.678	Bicylcoelemene	0.12
5	39.894	delta-Elemene	0.68
6	40.681	alpha-Cubebene	1.05
7	42.144	alpha-Ylangene	0.16
8	42.608	alpha-Copaene	5.77
9	43.349	beta-Cubebene	0.55
10	43.461	beta-Elemene	1.40
11	44.349	Cyperene	0.43
12	45.093	cis-alpha-Bergamotene	0.14
13	45.624	beta-Caryophyllene	49.24
14	46.007	gamma-Elemene	2.14
15	46.284	trans-alpha-Bergamotene	7.94
16	46.594	Aromadendrene	0.16
17	46.710	cis-beta-Farnesene	0.32
18	46.929	beta-Humulene	0.12
19	47.131	epi-beta-Santalene	0.14
20	47.251	trans-Muurola-3,5-diene	0.06
21	47.446	trans-beta-Farnesene	0.56
22	47.706	alpha-Humulene	7.22
23	47.935	allo-Aromadendrene	0.45
24	48.060	cis-Muurola-4(14),5-diene	0.03
25	48.395	Elemene isomer	0.06
26	48.667	cis-Cadina-1(6),4-diene	0.15
27	48.865	trans-Cadina-1(6),4-diene	1.89
28	49.008	gamma-Curcumene	0.13
29	49.273	Germacrene D	5.54
30	49.403	cis-cis-alpha-Farnesene	0.76
31	49.591	delta-Selinene	0.07
32	49.727	beta-Selinene	0.51
33	49.924	trans-Muurola-4(14),5-diene	0.44
34	50.136	Bicyclogermacrene	0.93
35	50.298	alpha-Muurolene	0.69
36	50.406	cis-alpha-Bisabolene	0.32
37	50.579	beta-Cadinene	0.29
38	50.883	beta-Bisabolene	3.07
39	51.062	cis-gamma-Bisabolene	0.23
40	51.181	gamma-Cadinene	0.64
41	51.338	Copaiba Sesquiterpenoid 1	0.08
42	51.506	delta-Cadinene	2.49
43	51.670	trans-Calamenene	0.05
44	51.808	beta-Sesquiphellandrene	0.56
45	51.991	trans-gamma-Bisabolene	0.06
46	52.335	trans-Cadina-1,4-diene	0.12
47	52.593	alpha-Cadinene	0.19
48	52.799	trans-alpha-Bisabolene	0.44
49	53.906	Germacrene B	0.23
50	54.549	Maaliol	0.06
51	54.887	Caryophyllene alcohol	0.24
52	55.268	Caryophyllene oxide	0.12
53	56.589	Ledol	0.03
54	57.510	Widdrol isomer	0.04
55	57.611	Humulane-1,6-dien-3-ol	0.26
56	57.904	1-epi-Cubenol	0.04
57	58.685	alpha-Muurolol	0.04
58	58.793	epi-alpha-Muurolol	0.10
59	58.946	delta-Cadinol	0.12
60	59.446	alpha-Cadinol	0.12
			100.00
