# Supplementary material for: Drosophila melanogaster as a model for studies related to the toxicity of lavender, ginger and copaiba essential oils
Source: PLoS One. 2023 Sep 28;18(9):e0291242. doi: 10.1371/journal.pone.0291242 (PMC10538661; doi:10.1371/journal.pone.0291242)
Supplement: S2 Dataset — Chromatographic analysis of the ginger essential oil utilized in this study, including the lot number, chromatographic chart, and a table of the constituents of the oil. (PDF) [file pone.0291242.s002.pdf]

# Aromatic Plant Research Center

## We provide uncompromising quality control for your products.

**Customer:** doTERRA International
**Lot Number:** 2183052
**Date Filled:** 43404

**Column:** ZB5 (60 m length × 0.25 mm inner diameter × 0.25 µm film thickness)
**Instrument:** Shimadzu GCMS-QP2010 Ultra
**Carrier gas:** Helium 80 psi
**Temperature ramp:** 2 degrees celsius per minute up to 260-degrees celsius
**Split ratio:** 30:1
**Sample preparation:** 5%w/v solution with Dichloromethane.

**Comments:**
The analysis of this GINGER lot revealed no contaminants or adulteration.
The sample meets the expected chemical profile for authentic essential oils of *Zingiber officinale*.

# GINGER  Essential Oil

**Customer:** doTERRA International
**Lot Number:** 2183052
**Date Filled:** 43404

## Chromatogram

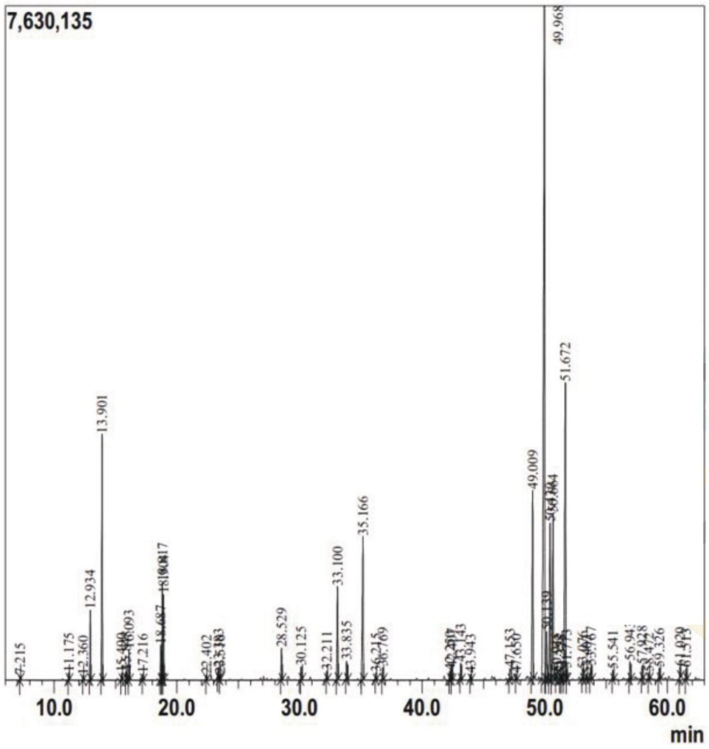


## Peak Report

| Ret.Time | Compound Name | Area % |
|---|---|---|
| 7.215 | Hexanal | 0.08 |
| 11.175 | 2-Heptanol | 0.15 |
| 12.36 | Tricyclene | 0.09 |
| 12.934 | alpha-Pinene | 1.6 |
| 13.901 | Camphene | 5.97 |
| 15.49 | beta-Pinene | 0.16 |
| 15.848 | 6-methyl-Hept-5-en-2-one | 0.21 |
| 16.093 | Myrcene | 0.75 |
| 17.216 | alpha-Phellandrene | 0.14 |
| 18.687 | Limonene | 1.02 |
| 18.817 | beta-Phellandrene | 2.76 |
| 18.904 | 1.8-Cineole | 2.16 |
| 22.402 | Terpinolene | 0.12 |
| 23.383 | 2-Nonanone | 0.29 |
| 23.51 | Linalool | 0.11 |
| 28.529 | Borneol | 0.99 |
| 30.125 | alpha-Terpineol | 0.39 |
| 32.211 | Citronellol | 0.24 |
| 33.1 | Neral | 3.12 |
| 33.835 | Geraniol | 0.56 |
| 35.166 | Geranial | 5.31 |
| 36.215 | Bornyl acetate | 0.18 |
| 36.769 | 2-Undecanone | 0.36 |
| 42.25 | alpha-Copaene | 0.27 |
| 42.407 | Geranyl acetate | 0.34 |
| 43.143 | beta-Elemene | 0.51 |
| 43.943 | Sesquithujene | 0.15 |
| 47.153 | trans-beta-Farnesene | 0.55 |
| 47.65 | Alloaromadendrene | 0.14 |
| 49.009 | Ar-Curcumene | 7.07 |
| 49.968 | alpha-Zingiberene | 34.19 |
| 50.139 | alpha-Muurolene | 1.79 |
| 50.43 | trans-trans-alpha-Farnesene | 5.52 |
| 50.664 | beta-Bisabolene | 6.13 |
| 50.915 | gamma-Cadinene | 0.19 |
| 51.258 | delta-Cadinene | 0.1 |
| 51.375 | alpha-Panasinsen | 0.16 |
| 51.672 | beta-Sesquiphellandrene | 12.33 |
| 51.773 | trans-gamma-Bisabolene | 0.24 |
| 53.076 | alpha-Elemol | 0.26 |
| 53.403 | cis-Sesquisabinene hydrate | 0.12 |
| 53.767 | trans-Nerolidol | 0.61 |
| 55.541 | trans-Sesquisabinene hydrate | 0.29 |
| 56.943 | Zingiberenol | 0.59 |
| 57.928 | 7-epi-cis-sesquisabinene hydrate | 0.43 |
| 58.472 | Zingerone | 0.18 |
| 59.326 | alpha-Eudesmol | 0.37 |
| 61.02 | 7-epi-trans-sesquisabinene hydrate | 0.34 |
| 61.513 | 2-cis-6-cis-Farnesol | 0.37 |

Area Total: **100**

**Comments:**
The analysis of this GINGER  lot revealed no contaminants or adulteration.
The sample meets the expected chemical profile for authentic essential oils of *Zingiber officinale*.
