# Supplementary material for: Drosophila melanogaster as a model for studies related to the toxicity of lavender, ginger and copaiba essential oils
Source: PLoS One. 2023 Sep 28;18(9):e0291242. doi: 10.1371/journal.pone.0291242 (PMC10538661; doi:10.1371/journal.pone.0291242)
Supplement: S3 Dataset — Chromatographic analysis of the lavender essential oil utilized in this study, including the lot number, chromatographic chart, and a table of the constituents of the oil. (PDF) [file pone.0291242.s003.pdf]

Aromatic Plant Research Center

We provide uncompromising
quality control for your products.

Customer : doTERRA International
Lot Number : 2210191
Date Filled : 01/19/2021

Column : ZB5 (60 m length × 0.25 mm inner diameter × 0.25 µm film thickness)
Instrument : Shimadzu GCMS-QP2010 Ultra
Carrier gas : Helium 80 psi
Temperature ramp : 2 degrees celsius per minute up to 260-degrees celsius
Split ratio : 30:1
Sample preparation : 5%w/v solution with Dichloromethane.

Comments:
The analysis of this Lavender lot revealed no contaminants or adulteration.
The sample meets the expected chemical profile for authentic essential oils of Lavandula angustifolia.

Analysied by : Ambika Poudel
Reviewed by : Dr. Prabodh Satyal

Aromatic Plant Research Center
230 N 1200 E STE 100
Lehi, UT 84043
www.aromaticplant.org

Lavender Essential Oil

Customer : doTERRA International

Lot Number : 2210191

Date Filled : 01/19/2021

Chromatogram

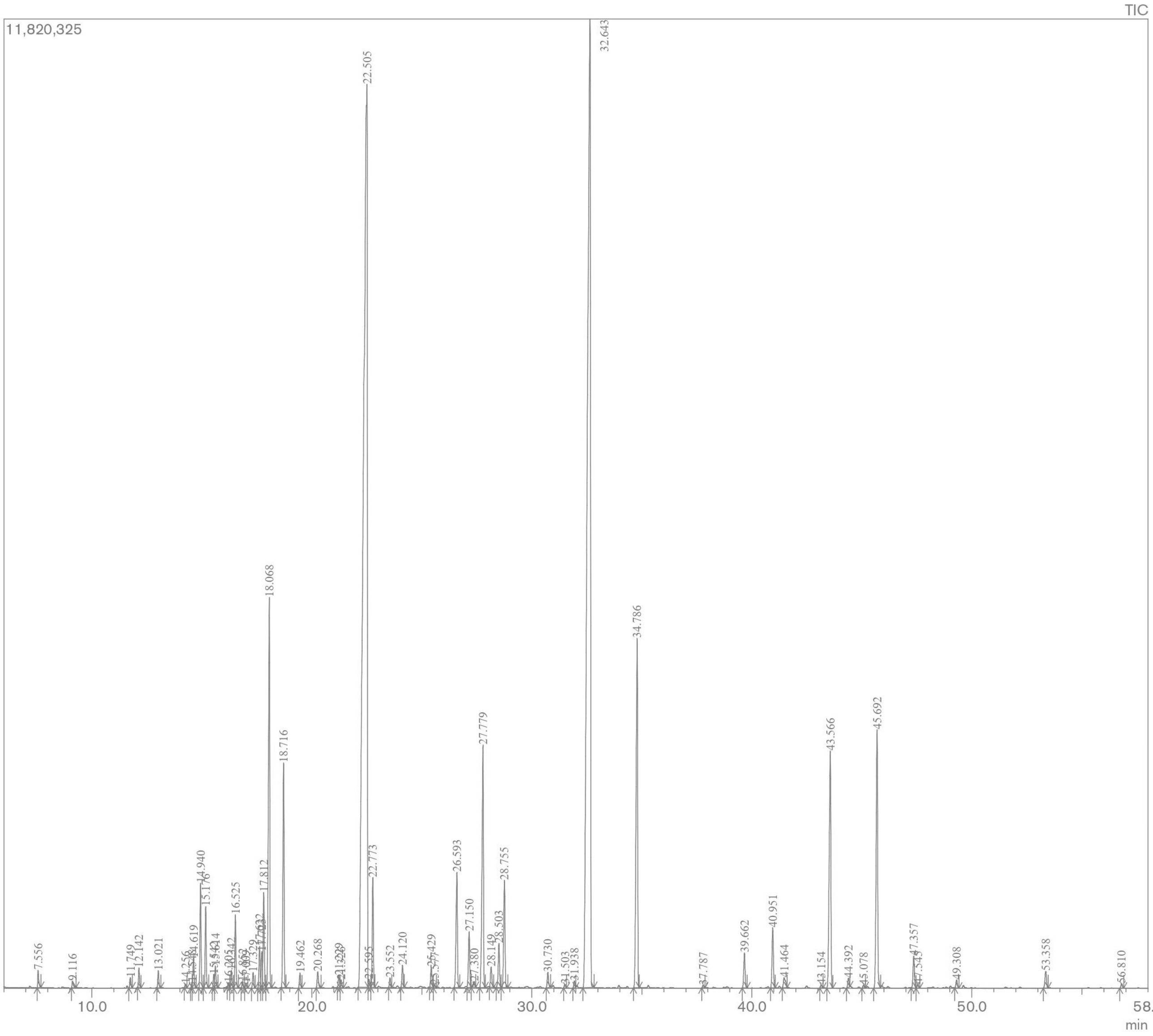

Aromatic Plant Research Center
230 N 1200 E STE 100
Lehi, UT 84043
www.aromaticplant.org

Peak Report

Peak#	R.Time	Name	Area%
1	7.556	1-Methoxy hexane	0.15
2	9.116	n-Hexanol	0.08
3	11.749	alpha-Thujene	0.10
4	12.142	alpha-Pinene	0.19
5	13.021	Camphene	0.18
6	14.256	Sabinene	0.03
7	14.548	beta-Pinene	0.04
8	14.619	1-Octen-3-ol	0.33
9	14.940	3-Octanone	1.05
10	15.176	Myrcene	0.82
11	15.542	Butyl butanoate	0.13
12	15.614	3-Octanol	0.23
13	16.205	alpha-Phellandrene	0.04
14	16.342	delta-3-Carene	0.18
15	16.525	Hexyl acetate	0.76
16	16.852	alpha-Terpinene	0.06
17	17.009	meta-Cymene	0.05
18	17.329	para-Cymene	0.17
19	17.622	Limonene	0.46
20	17.723	beta-Phellandrene	0.38
21	17.812	1,8-Cineole	0.99
22	18.068	cis-beta-Ocimene	4.71
23	18.716	trans-beta-Ocimene	2.49
24	19.462	gamma-Terpinene	0.16
25	20.268	cis-Linalool oxide (furanoid)	0.20
26	21.229	Terpinolene	0.13
27	21.326	trans-Linalool oxide (furanoid)	0.10
28	22.505	Linalool	33.03
29	22.595	Hexyl propionate+Hotrienol	0.09
30	22.773	1-Octen-3-yl acetate	1.15
31	23.552	3-Octanol acetate	0.10
32	24.120	allo-Ocimene	0.24
33	25.429	Camphor	0.25
34	25.577	4-Isopropyl-3-cyclohexen-1-one	0.03
35	26.593	Lavandulol	1.49
36	27.150	Borneol	0.67
37	27.380	3-trans-5-cis-1,3,5-Undecatriene	0.07
38	27.779	Terpinen-4-ol	3.28
39	28.149	Cryptone	0.25
40	28.503	Hexyl butanoate	0.54
41	28.755	alpha-Terpineol	1.35
42	30.730	Nerol	0.19
43	31.503	Hexyl 2-methyl butanoate	0.04
44	31.938	Cumin aldehyde	0.09
45	32.643	Linalyl acetate	28.20
46	34.786	Lavandulyl acetate	5.00
47	37.787	Hexyl tiglate	0.03
48	39.662	Neryl acetate	0.42
49	40.951	Geranyl acetate	0.73
50	41.464	Hexyl hexanoate	0.19
51	43.154	cis-alpha-Bergamotene	0.04
52	43.566	beta-Caryophyllene	3.66
53	44.392	trans-alpha-Bergamotene	0.11
54	45.078	cis-beta-Farnesene	0.03
55	45.692	trans-beta-Farnesene	3.66
56	47.357	Germacrene D	0.41
57	47.545	trans-beta-Bergamotene	0.05
58	49.308	gamma-Cadinene	0.10
59	53.358	Caryophyllene oxide	0.23
60	56.810	epi-alpha-Cadinol	0.06
			100.00
