# Supplementary material for: Drosophila melanogaster as a model for studies related to the toxicity of lavender, ginger and copaiba essential oils
Source: PLoS One. 2023 Sep 28;18(9):e0291242. doi: 10.1371/journal.pone.0291242 (PMC10538661; doi:10.1371/journal.pone.0291242)
Supplement: Attachment — Submitted filename: Comments and questions for manuscript (PONE-D-23-11609).docx [file pone.0291242.s004.docx]

I have reviewed the paper titled: *Drosophila melanogaster* as a model for studies related to the toxicity of lavender, ginger and copaiba essential oils. The topic of the manuscript is within the scope of the Journal. The topic of research is interesting, but there are some problems in the manuscript. Following are the issues that may be helpful for the authors to improve this manuscript. The comments below should be considered.

**Comments regarding the manuscript are as follows:**

1) Should be checked in italics and abbreviations in the manuscript. Abbreviations: At the first appearance in the abstract and the text, abbreviations should be preceded by words for which they stand.
2) Maybe authors should be added the keywords such as; *genotoxicity, environment and health, risk assessment,* etc.
3) I think that the concentrations of essential oils (EOs) of copaiba (*Copaifera sp.*), ginger (*Zingiber officinale*), and lavender (*Lavandula angustifolia*) (döTerra, Utah, USA) should be added to the Abstract.
4) Introduction: Authors should discuss the meaningfulness using *Drosophila melanogaster* compared to other *in vivo* model organisms. The flow of introduction need to be tuned.
5) The reason for using the *in vivo* model? What was the rationale aim for choosing *D. melanogaster* as a model organism? This information must be included in the Introduction section.
6) What is the significance of investigating EOs exposure routes?
7) The researchers need to recommend the dosage range which will be safe for human consumption.
8) I think that it should be mentioned in the Materials and Methods to reference studies for selected concentrations of EOs (v/v)? I think this should be explained and added in the manuscript in order to enable comparisons to other studies. How could they justify the concentrations selection for the study? Are these low or high concentrations? Working with high concentrations reduces the toxicological value of the results since they select the resistant individuals, or simply do not occur in real life. Is the dose limited by toxicity? The authors mention that in the Materials and methods.
9) The Materials and methods section should be referenced from the literature.
10) What's the likelihood for human exposure to used concentrations in EOs? Are they realistic exposure concentrations? Please explain the experimental rationale in discussion section. Please define better the relation between the tested dose and the potential environmental exposure. Moreover, the discussion should be improved with a more stressed comparative analysis of the own research results with literature works, already present.
11) The activation of toxicity mechanisms are not completely clear, and miss of any deep investigation.
12) What were the rationale for choosing the different *in vivo* assays? This must be included in the Introduction section.
13) How many independent experiments (excluding for replicates) were performed in this study? I think the authors should mention in the Materials and Methods section. Application period projected for groups were chosen according to what? The authors should fully clarify this point.
14) Section Discussion: This section also need to be improved with recent studies. i feel that some irrelevant discussion. Please discuss about the effect of EOs on *D. melanogaster* as an *in vivo* model.
15) What is the vehicle control name? Which were used group in the Statistical analysis?
16) What is positive control used for the assays? I think the authors should add values of positive control in the Figures.
17) The correlation between selected compound (or the viability) and the *in vivo* experiments?
18) In addition in the discussion section comparative evaluation with toxicological effects at cellular levels of EOs could add additional relevance to the work. Please define better the relation between the tested concentration and the potential environmental exposure. Moreover, the discussion should be improved with a more stressed comparative analysis of the own research results with literature works, already present.
19) The authors should include in the discussion some thoughts about how the *in vivo* findings can translate to human risk.
20) The authors should be added chemical constituents (%) (such as, CAS No, Quality %, Catalog number, etc.)
21) use abbreviations for SI units: d for day.
22) What do authors think about the effects of solubility of EOs on the cytotoxicity?
23) The authors should add some description about the disadvantages or difficulties of using animal *in vivo* and *in vitro* models related with EOs.
24) In Discussion: The authors should add new papers and update the literature review. More evidence on the link between toxicity and intestinal damage/life span caused by EOs could support the paper's idea (novelty) better.
25) The rationale for the selection of EOs must be stated at the end of the Discussion section.
26) Running title should be added.
27) In Figure legends, please indicate the following in each Figure legend: (a) the times of each experiments were repeated; (b) the number/replica of each group in each repeat; (c) are the data showed in the figure representative?
28) Please highlight the novelty aspect of the present research in the introduction, abstract and conclusion section.
29) Statistical analysis: The authors need to provide company of stats for the statistical software. There is not enough explanation in statistical analysis. What statistical tests were used in the manuscript? Arithmetic mean ± standard deviation (or arithmetic mean ± standard error) values should be shown in the Figures.
30) What were the rationale for choosing the different assays? This must be included in the Introduction section. Do the assays used by the authors are based on the OECD guidelines?
31) Reported images are really poor and need substantial improvement.
